# Speech Perception in Noise Is Associated With Different Cognitive Abilities in Chinese-Speaking Older Adults With and Without Hearing Aids

**DOI:** 10.3389/fpsyg.2021.640300

**Published:** 2022-01-04

**Authors:** Yuan Chen, Lena L. N. Wong, Shaina Shing Chan, Joannie Yu

**Affiliations:** ^1^Department of Special Education and Counseling, The Education University of Hong Kong, Tai Po, Hong Kong SAR, China; ^2^Clinical Hearing Sciences (CHearS) Laboratory, Faculty of Education, The University of Hong Kong, Pokfulam, Hong Kong SAR, China; ^3^Audiology Centre, Union Hospital, Tai Wai, Hong Kong SAR, China

**Keywords:** cognitive function, hearing loss, speech perception, older adults, Chinese

## Abstract

Chinese-speaking older adults usually do not perceive a hearing problem until audiometric thresholds exceed 45 dB HL, and the audiometric thresholds of the average hearing-aid (HA) user often exceed 60 dB HL. The purpose of this study was to examine the relationships between cognitive and hearing functions (measured as audiometric or speech reception thresholds) in older Chinese adults with HAs and with untreated hearing loss (HL). Participants were 49 Chinese older adults who used HAs and had moderate to severe HL (HA group), and 46 older Chinese who had mild to moderately severe HL but did not use HAs (untreated; or UT group). Multiple linear regression analysis was employed to evaluate how well age, education level, audiometric thresholds, and speech perception in noise were related to performance on general cognitive function, working memory, executive function, attention, and verbal learning tests. Results showed that speech perception in noise alone accounted for 13–25% of the variance in general cognitive function, working memory, and executive function in the UT group, and 9–21% of the variance in general cognitive function and verbal learning in the HA group (i.e., medium effect sizes). Audiometric thresholds did not explain any proportion of the variance in cognitive functioning in the HA or UT group. Thus, speech perception in noise accounts for more variance in cognitive performance than audiometric thresholds, and is significantly associated with different cognitive functions in older Chinese adults with HAs and with untreated HL.

## Introduction

### Hearing Loss and Hearing Aid Uptake in Chinese-Speaking Older Adults

Hearing loss (HL) is reported in nearly two-thirds of adults aged 60 years and older (Gong et al., 2018). However, Chinese older adults often do not report hearing problems until the HL exceeds 45 dB hearing level, and typically do not acquire a hearing aid (HA) until an average loss of about 65 dB hearing level, even though normal hearing sensitivity is defined as pure-tone audiometric thresholds not exceeding 25 dB HL (Doyle and Wong, 1996). Similarly, studies by the Institute of Human Communicative Research (2005) and Wong et al. (2014) found that the majority of participants with untreated HL exhibit mild to moderately severe audiometric HL, and those with HAs exhibit moderate to severe HL. The HA uptake rate is lower among older Chinese speakers with HL (less than 10%) than the rate of approximately 25% reported in the United States and United Kingdom (Chien and Lin, 2012; Zhao et al., 2015; Bisgaard and Ruf, 2017).

### The Relationship Between Hearing Loss and Cognitive Function

Epidemiological studies suggest that HL in older adults is independently associated with an increased incidence of cognitive decline, after accounting for factors such as age, gender, income, education, general physical health, and HA use (Lin et al., 2011, 2013; Gurgel et al., 2014; Harrison Bush et al., 2015). Specifically, older adults with HL experience a 30–40% faster cognitive decline than that in the general older population (Lin et al., 2013). Cross-sectional and longitudinal studies have shown an association between HL and cognitive decline in general, as well as in specific domains such as verbal learning (VL), sustained attention (SA), executive function (EF), and working memory (WM) in older adults (Wingfield et al., 2005; Pichora-Fuller and Singh, 2006; Arlinger et al., 2009; Lin et al., 2011, 2013). In a meta-analysis, Taljaard et al. (2016) found a medium effect in individuals with treated HL, and a small effect in those with untreated HL, when evaluating the relationship between HL and general cognitive function (GCF). However, some studies, such as the one by Harrison Bush et al. (2015), reported very small effect sizes or were underpowered in relating HL to cognitive function, while others did not find any such relationship (Gallacher, 2005).

As better education contributes to better cognitive functioning in older adults (Livingston et al., 2017), cognitive decline is of particular concern for Chinese speakers aged 60 years and above, as their median education level is 5.89 years (National Bureau of Statistics in China, 2011). This is much lower than that of HA users in studies on the relationship between hearing and cognitive functions conducted in Western societies (Lin et al., 2011; Dawes et al., 2015; Maharani et al., 2018).

### Measurement of Hearing Function

The weak association between HL and cognitive functioning may be attributed to the use of pure-tone audiometric thresholds as an indicator of hearing function (i.e., the ability to perceive sounds) (Plack, 2014). Audiometric hearing thresholds primarily reflect peripheral hearing impairment and may not reflect auditory cortical processing (Tun et al., 2012). In a large-scale study involving a 60-h battery of various tests of cognitive and auditory functioning, Humes et al. (2013) found that decreased auditory sensory functioning, which was a composite of non-speech psychophysical and speech perception measures, was associated with reduced cognitive functioning, and the authors concluded that relying on audiometric thresholds alone may underestimate the relationship between hearing and cognitive functions. Therefore, being able to recognize speech in noise, which involves not only peripheral hearing but also higher auditory cortical processing, and reflects daily listening ability (Humes et al., 2013), may be a better indicator of a decline in auditory sensory functioning.

Different degrees of HL (i.e., ranging from mild to profound HL) were mixed and whether HA was fitted were not specified in previous studies, rendering comparisons in findings across studies difficult (Harrison Bush et al., 2015; Taljaard et al., 2016). More severe HL is associated with poorer suprathreshold auditory processing skills, which could lead to greater temporal information distortion in speech (Füllgrabe and Moore, 2018). Similarly, HA processing that alters certain acoustic information to enhance hearing could introduce spectral and temporal distortions (Stone et al., 2009a; Wong et al., 2018). Such distortions could disrupt automatic lexical retrieval, leading to explicit, effortful processing mechanisms that rely on cognitive processing system (Rönnberg et al., 2019). Thus, when analyzing the relationship between HL and cognitive functioning, individuals with different degrees of HL should be considered separately. Similarly, individuals using HAs should not be treated in the same way as those who do not.

Therefore, the first aim of the current study was to examine whether pure-tone audiometric and speech reception thresholds were significantly related to cognitive function in two groups: older adult HA users with moderate to severe HL, and older adults with untreated mild to moderately severe HL. These two groups represent typical older HA users and non-HA users in Hong Kong and mainland China (Wong et al., 2014), for whom the relationship between hearing and cognitive functioning has not been studied. Findings from the literature may not apply to Chinese speakers, as typical HA users in Western societies exhibit different characteristics (i.e., higher education level and a higher proportion of HA uptake, especially in older adults with mild HL) (Doyle and Wong, 1996).

Furthermore, the causal mechanisms underlying the link between auditory sensory and cognitive decline is still unclear. Wayne and Johnsrude (2015) reviewed several potential mechanisms. Among them, three hypotheses have emerged as strong contenders: (1) the information-degradation hypothesis, which suggests that auditory sensory decline leads to impoverished (but reversible) cognitive function; (2) the sensory deprivation hypothesis, which indicates that auditory sensory decline causes more permanent cognitive decline; and (3) the common cause hypothesis, which suggests that a third variable contributes to declines in auditory sensory and cognitive functions. While it is difficult to separately test these hypotheses, it is important to know which cognitive skill is significantly related to hearing function. Therefore, the second aim of the current study was to examine which cognitive skill was related to audiometric thresholds and speech perception in the two participant groups.

There are three differences between the current and previous studies examining the relationship between audiometric thresholds, speech perception and cognitive function (e.g., Akeroyd, 2008; Humes et al., 2013). First, as mentioned above, cognitive functions of HA users and non-users were examined separately. Second, instead of using speech perception as a dependent variable (e.g., Humes, 2007; Lunner and Sundewall-Thorén, 2007), the current study used cognitive function test scores as dependent variables, with speech perception and audiometric thresholds as independent variables, while controlling for the effects of age, peripheral HL, and education level. Accordingly, the results indicated which cognitive function(s) were more likely associated with auditory sensory decline in the UT and HA groups. Third, although examining speech perception and cognitive function in a pre-post design (i.e., HA users before and after fitting) may be better at controlling confounders, the untreated comparison group (i.e., HA users before fitting) would not represent the largest untreated HL population in China, as Chinese older adults typically do not acquire HAs until an average loss of about 65 dB is reached (Doyle and Wong, 1996). In contrast, the two independent samples included in the present study represent the largest relevant populations in China: the untreated HL group, representing those with mild to moderately severe audiometric HL, and the HA group, representing those with moderate to severe HL.

## Materials and Methods

### Participants

A convenience sample of 50 current HA users with moderate to severe HL (i.e., the HA group), and 50 untreated participants (non-HA users) with mild to moderately severe HL (i.e., the UT group) were recruited from the Audiological Center at the Prince of Wales Hospital and Alice Ho Miu Ling Nethersole Hospital, in Hong Kong. Participants in the HA group must have worn HAs for at least 2 years. This restriction was employed because it normally takes 2–5 years for acclimatization to HAs (Ng and Rönnberg, 2020). All participants were required to be older than 60 years and speak Cantonese. All had normal or corrected-to-normal vision as screened using the Snellen Chart. Patients were excluded if they had a history, as documented in medical records, of neurodegenerative disorders, brain tumors, significant head trauma, epilepsy, significant psychiatric disorders (such as major depression or schizophrenia), substance abuse, or alcoholism.

### Materials

Hearing thresholds were obtained in a standard audiometric booth at the Audiology Center of the Prince of Wales Hospital using a GSI 61 Audiometer (Grason-Stadler, Eden Prairie, MN, United States). Aided and unaided soundfield hearing thresholds at 0.5, 1, 2, and 4 kHz were measured binaurally using warble tones, in the same room, using the GSI61 Audiometer, and were used to determine the severity of HL with and without HAs. Sounds were presented via a loudspeaker (Cerwin-Vega) located 1 m in front of the participant. In addition, because soundfield hearing thresholds cannot determine the better/worse ear for SRT testing, ear-specific air-conduction thresholds at 0.25, 0.5, 1, 2, 4, and 8 kHz were obtained using TDH-49 headphones.

Speech reception thresholds (SRTs), defined as the presentation level at which 50% of sentences were repeated entirely correctly by the participant (Wong and Soli, 2005), were obtained using the Cantonese version of the Hearing in Noise Test (CHINT) (Wong and Soli, 2005) in four test conditions: quiet (SF), with noise originating from the front (noise front: NF), on the side of the better ear (noise better ear: NBE), and on the side of the worse ear (noise worse ear: NWE). Each test condition included 20 sentences. Speech was always presented at 0° azimuth using a loudspeaker, and the intensity level of the sentences was adjusted adaptively, depending on the correctness of the response. Speech-spectrum shaped noise was used as a masker and fixed at 65 dB A in the noise conditions. SRTs in the quiet condition were measured in dB A, with lower SRT suggesting better speech perception in quiet. SRTs in noise were measured as signal-to-noise ratios (SNR), with lower SRTs suggesting better ability to extract speech from noise. The starting level was 65 and +5 dB SNR for quiet and noise conditions, respectively. The step size was 4 dB for the first four sentences and 2 dB for the 5th to 20th sentences. A noise composite score was calculated to represent the overall performance in noise, and was based on the performance when noise was from the front and from the side using the following formula: [NF + ½ (NBE + NWE)]/2.

The Cantonese version of the Montreal Cognitive Assessment (MoCA; Wong et al., 2009), and four tests selected from the CogState Battery (CSB),^1^ were used to assess cognitive functioning.

The MoCA, covering eight domains of cognitive function: attention and concentration, EF, memory, language, visuospatial skills, conceptual thinking, calculations, and orientation, was designed to assess GCF and detect mild cognitive impairment (MCI). The test takes 10 mins to administer and the total score ranges from 0 to 30, with higher scores indicating better cognitive functioning. The Cantonese Chinese version was retrieved from the official website^2^. It has been adapted into Chinese with good reliability and validity (Zhong et al., 2013). It has been validated in Chinese older adults in Hong Kong, and was determined to be brief and feasible for administration in clinical settings (Yeung et al., 2014). Although the MoCA is often used to detect MCI, no exclusion of participants was made based on the MoCA score. This was because individuals with HL tend to have a higher risk of cognitive impairment than those without HL (Fritze et al., 2016). The relationship between HL and cognitive function may be obscured if participants with MCI were excluded.

The four selected CSB subtests comprised the One-Back Test (OBT), Groton Maze Learning Test (GML), Identification Test (IDN), and International Shopping List Task (ISL), which were used to examine WM, EF, SA, and VL, respectively. The CSB test battery has been shown to be sensitive in detecting MCI and Alzheimer’s disease in older adults (de Jager et al., 2009).

The OBT was used to evaluate WM. A series of playing cards were presented one-by-one in the middle of a computer screen. Participants were requested to identify whether the playing card presented on the screen was the same as the previous one by pressing the “yes” button (the right button on the mouse) or the “no” button (the left button). Participants were encouraged to work as quickly as they could and be as accurate as possible. Accuracy of performance is reported as the arcsine transformation of the square root of the proportion of correct responses. Higher scores represent better performance. This test was relatively easy compared to other WM tests (e.g., the Reading Span Test). It was the only non-verbal WM test that has been validated in Cantonese speakers during the data collection, and Chen et al. (2020) demonstrated a lack of ceiling effects in a group of Chinese-speaking older adults with HAs (Chen et al., 2020).

The GML was used to evaluate EF. Participants were asked to learn the same hidden pathway in a maze on five consecutive trials. This learning process requires goal-directed problem-solving skills including set shifting, WM, and inhibitory control, which are important elements of EF (Carlson et al., 2013). The total number of errors made in attempting to learn the same hidden pathway is reported, and higher scores indicate poorer EF.

The IDN was used to evaluate SA. A playing card was presented face down in the center of the screen. Participants were asked to press the “yes” button if the card was red when flipped over, and press the “no” button if the card was black when flipped over. Participants were asked to complete the trials as fast and accurately as possible. The speed of performance is reported as the mean of the log10 transformed reaction times for correct responses. Lower scores represent better performance.

The ISL was developed to assess VL in populations with diversity in language and cultural backgrounds. A total of 12 food items were presented on a screen facing the test administrator (a trained research assistant) who read the words one by one to the participants. Participants were instructed to repeat each word one by one, along with the administrator, to ensure that all words were intelligible to them. Subsequently, the participants were asked to recall as many of the items as they could. The same list and procedure were repeated two more times. The total number of items from the list that were correctly recalled were summed to compute the score; thus, higher scores indicate better performance.

### Procedures

This was a cross-sectional and observational study with two distinct groups of participants. Ethical clearance was obtained from the University of Hong Kong and Chinese University of Hong Kong. After an explanation of the nature of the study and procedures, written consent was obtained from participants. Demographic information was collected from all participants, followed by hearing assessments. HA verification and fine-tuning were conducted by audiologists to ensure the best fit before testing. During the actual testing, the MoCA, OBT, GML, IDN, and ISL were administered according to the instructions provided in the manuals. The order of SRT testing in quiet and noise conditions was randomized across participants. SRTs were obtained with sentence lists randomly selected by the CHINT program. Participants were encouraged to make guesses even when unsure. A pocket-talker was fitted to participants in the UT group and the volume was set to a comfortable listening level to ensure good reception of the test instructions, and test stimuli of the ISL. Participants in the HA group set HAs to their usual settings during the cognitive assessment. Verbal instructions were repeated and participants’ understanding of the instructions was checked by asking them to verbally recall the instructions prior to the administration of each test. Each test (except the MoCA) started with a practice session.

The entire testing session took approximately 2 h. To avoid fatigue, a break was given to participants after 1 h of assessment or upon request. A transportation allowance of HKD 200 (equivalent to USD 25) was provided to each participant.

### Data Analysis

Independent samples *t*-tests or Mann-Whitney *U* tests were used to examine whether there were significant differences in audibility and SRTs between two groups. Shapiro Wilk tests were used to examine whether data were normally distributed. Multiple linear regression analyses using a forward method were employed to evaluate how well age, education level, soundfield hearing thresholds (unaided soundfield hearing thresholds for the UT groups and aided soundfield hearing thresholds for the HA group), and SRTs in noise were associated with performance in each cognitive domain (i.e., GCF, EF, SA, WM, and VL). Education level was coded as 0 (uneducated), 1 (primary school), 2 (secondary school), and 3 (tertiary school or above). Pearson product-moment correlation coefficients were calculated to examine the correlation among variables before conducting multiple linear regression analysis. Data analyses were conducted using IBM SPSS Statistic 21.0 for Windows (IBM Corp., Armonk, NY, United States).

## Results

### Demographics and Hearing Assessment

Due to scheduling conflicts, 49 participants in the UT group and 46 participants in the HA group completed all tests. All the following analysis were based on these participants. Most HA participants (94%) were unilaterally fitted with a HA, while the remaining were bilaterally fitted (Table 1). The mean duration of HA use was 7.28 years (SD = 4.77; range: 2–20 years). Participants were wearing different brands of HAs including Resound (*n* = 15), Beltone (*n* = 19), Widex (*n* = 4), Phonak (*n* = 4), Oticon (*n* = 1), and Siemens (*n* = 3). All participants were using their HAs at least 3–4 days/week, with 33 using their HA every day. For 11/46, 14/46, and 20/46 participants, HA use was less than 4, 4–8 h, and more than 8 h per day, respectively.

**TABLE 1:** Demographic characteristics, speech recognition thresholds, and cognitive functions of the untreated (UT) and hearing aid (HA) groups.

	UT group	HA group
Mean age in years (SD) [range]	71.41 (6.33) [61–87]	68.74 (5.05) [60–83]
Gender (%male/%female)	51%/49%	41%/59%
**Education level**		
Uneducated (0 years)	14%	0%
Primary (1–6 years)	43%	63%
Secondary (7–13 years)	41%	37%
Tertiary (>13 years)	2%	0%
**Hearing level**		
Unaided soundfield hearing thresholds at 0.5, 1, 2, and 4 kHz in dB HL (SD) [range]	47.50 (10.69) [27.50–67.5]	64.89 (10.73) [41.25–88.75]
Aided bilateral soundfield hearing thresholds at 0.5, 1, 2, and 4 kHz in dB HL (SD) [range]	N/A	47.96 (8.30) [28.75–65.00]
**SRT**		
Quiet in dBA (SD)	56.16 (10.90) (unaided)	61.49 (9.40) (aided)
Noise from front in dB SNR (SD)	3.29 (4.34) (unaided)	5.67 (4.02) (aided)
Noise from better ear side in dB SNR (SD)	3.13 (4.99) (unaided)	7.23 (4.98) (aided)
Noise from worse ear side in dB SNR (SD)	0.89 (5.29) (unaided)	4.66 (5.11) (aided)
*Noise composite in dB SNR (SD)	2.69 (4.52) (unaided)	5.87 (4.11) (aided)
**Cognitive assessment**		
MoCA, general cognitive function (SD)	23.86 (4.32)	23.85 (4.18)
OBT, working memory (SD)	1.13 (0.21)	1.09 (0.24)
GML, executive function (SD)	118.94 (77.12)	115.89 (57.61)
IDN, attention (SD)	2.81 (0.08)	2.81 (0.08)
ISL total, verbal learning (SD)	18.47 (3.85)	20.22 (4.53)
ISL trial 1 (SD)	4.22 (1.48)	4.93 (1.57)
ISL trial 2 (SD)	6.47 (1.76)	7.15 (1.73)
ISL trial 3 (SD)	7.78 (1.77)	8.13 (2.13)

*SD, standard deviation; N/A, not applicable; MoCA, Montreal Cognitive Assessment; OBT, One-Back Test; GML, Groton Maze Learning Test; IDN, Identification Test; ISL, International Shopping List Task; UT, untreated; HA, hearing aid; SNR, signal-to-noise ratio.*

*A noise composite score was calculated to represent the overall performance in noise, and was based on the performance when noise was from the front and from the side using the following formula: [NF + ½ (NBE + NWE)]/2. NF, noise from front; NBE, noise from the better ear side; NEW, noise from worse ear side.

Although an independent samples *t*-test [*t* (93) = −7.91, *p* < 0.001] showed the HA group exhibited a significantly worse unaided soundfield hearing threshold (mean = 64.89, SD = 10.73) compared to that in the UT group (mean = 47.50, SD = 10.69), there was no significant difference [independent *t*-test, *t* (93) = 0.23, *p* = 0.82] between the aided soundfield hearing threshold in the HA group (mean = 47.96, SD = 8.30) and the unaided soundfield hearing threshold in the UT group (mean = 47.50, SD = 10.69). These results suggest that although the HA group possessed more severe unaided HL than did the UT group, aided hearing thresholds in the HA group were comparable to the unaided hearing thresholds in the UT group (i.e., audibility) when measured in the soundfield. However, the UT group was significantly better at perceiving sentences in noise (mean = 2.69, SD = 4.52) than the HA group (mean = 5.87, SD = 4.10) [independent *t*-test, *t* (93) = −3.58, *p* = 0.001; see Table 1]. Furthermore, using a cutoff score of 22 for the Cantonese version of MoCA, as recommended by Yeung et al. (2014), 27% of participants in the UT group, and 26% of participants in the HA group, were regarded as exhibiting cognitive impairment.

### Factors Associated With Cognitive Functions

The Pearson product-moment correlation coefficients indicated that age was significantly correlated with all cognitive functions, with the exception of the GCF, in the UT group, and GCF, SA, and EF in the HA group. The soundfield hearing threshold was significantly correlated with GCF, WM, and EF in the UT group. Sentence perception in noise was significantly correlated with GCF, WM, EF in the UT group, and GCF, and VL in the HA group. Education level was correlated with all cognitive functions, with the exception of VL, in the UT group, and significantly correlated with EF in the HA group (Tables 2, 3).

**TABLE 2:** Correlations among variables in the UT group.

	Age	Education level	Unaided soundfield hearing thresholds	Sentence perception in noise	General cognitive function	sustained Attention	Working memory	Executive function
Education level	−0.36*							
Unaided soundfield hearing thresholds	0.54**	–0.28						
Sentence perception in noise	0.47**	–0.12	0.64**					
General cognitive function	–0.22	−0.41**	−0.29*	−0.41**				
Sustained attention	0.35*	−0.45**	0.01	0.18	0.42**			
Working memory	0.42**	0.45**	−0.40**	−0.51**	0.52**	–0.26		
Executive function	0.41**	−0.35*	0.30*	0.50**	−0.56**	0.40**	−0.56**	
Verbal learning	−0.39**	0.23	–0.23	–0.16	−0.29*	–0.25	0.20	-0.31*

*Correlation is significant at the 0.05 level (2-tailed).

**Correlation is significant at the 0.01 level (2-tailed).

*Correlation coefficients marked with bold indicate statistically significant relationships.*

**TABLE 3:** Correlations among variables in the HA group.

	Age	Education level	Aided soundfield hearing thresholds	Sentence perception in noise	General cognitive function	sustained Attention	Working memory	Executive function
Education level	–0.00							
Aided soundfield hearing thresholds	–0.04	–0.14						
Sentence perception in noise	0.26	–0.11	0.41**					
General cognitive function	–0.27	0.18	–0.14	−0.30*				
Sustained attention	0.12	–0.06	–0.01	0.21	–0.17			
Working memory	−0.32*	0.20	0.06	–0.27	0.42**	–0.21		.
Executive function	0.17	−0.33*	0.14	0.06	−0.37*	0.00	−0.42**	
Verbal learning	−0.31*	0.22	–0.05	−0.46**	0.31*	−0.34**	0.19	-0.18

*Correlation is significant at the 0.05 level (2-tailed).

**Correlation is significant at the 0.01 level (2-tailed). Correlation coefficients marked with bold indicate statistically significant relationships.

#### Untreated Group

Multiple linear regression analyses showed that SRTs in noise were significantly related to WM and EF, accounting for 25% of the variance of these cognitive functions (Table 4). In addition, when education level was included in these analyses, a further 8 and 14% of the variance in WM and EF could be further explained, respectively. Together, SRTs in noise and education level accounted for 30–39% of the variance in GCF, WM, and EF.

**TABLE 4:** Results from multiple linear regression analyses in the UT group.

Model	B	SE B	Beta	*R* ^2^
**General cognitive function (MoCA)**				
*Step 1*				0.17
Constant***	18.34	1.88		
Education level**	2.39	0.78	–0.41	
*Step 2*				0.30
Constant***	19.86	1.82		
Education level**	2.14	0.73	–0.37	
SRTs in noise**	–0.35	0.12	0.36	
**Working memory**				
*Step 1*				0.25
Constant***	1.19	0.03		
SRTs in noise***	–0.02	0.01	–0.51	
*Step 2*				0.39
Constant***	0.93	0.08		
SRTs in noise***	–0.02	0.01	–0.46	
Education level**	0.11	0.03	0.39	
**Executive function**				
*Step 1*				0.25
Constant***	96.06	11.26		
SRTs in noise***	8.52	2.16	0.50	
*Step 2*				0.33
Constant***	167.40	31.64		
SRTs in noise**	7.92	2.07	0.46	
Education level*	–30.24	12.62	–0.29	
**Sustained attention**				
*Step 1*				0.20
Constant***	2.92	0.03		
Education level**	–0.05	0.01	–0.45	
**Verbal learning**				
*Step 1*				0.15
Constant***	35.39	5.85		
Age**	–0.24	0.08	–0.39	

*Independent variables included age, education level, soundfield hearing thresholds, and SRTs in noise. Education level was coded as 0 = uneducated, 1 = primary school, 2 = secondary school, and 3 = tertiary school or above. UT, untreated; MoCA, Montreal Cognitive Assessment; SRT, speech reception threshold; B, raw coefficients; SE B, standard error of b; ΔR^2^, the change of R^2^ for each subsequent step.*

*p < 0.05, **p < 0.01, ***p < 0.001.

However, SRTs in noise were not significantly related to SA or VL, while 20% of the variance in SA was accounted for by education level alone. Finally, age was the only variable related to VL, accounting for 15% of the variance.

#### Hearing Aid Group

Speech reception thresholds in noise were significantly associated with GCF and VL, accounting for 9 and 21% of the variance in GCF and VL, respectively (Table 5). Education level was the only variable associated with EF, accounting for 11% of the variance. Age significantly contribute to the WM. No factor was significantly associated with SA in the HA group.

**TABLE 5:** Results from multiple linear regression analyses in the HA group.

Model	B	SE B	Beta	*R* ^2^
**General cognitive function (MoCA)**				
*Step 1*				0.09
Constant***	25.63	1.04		
SRTs in noise*	–0.31	0.15	-0.30	
**Working memory**				
*Step 1*				0.10
Constant***	2.19	0.46		
Age*	–0.02	0.01	-0.34	
**Executive function**				
*Step 1*				0.11
Constant***	207.68	40.65		
Education level*	–38.74	16.81	-0.33	
**Sustained attention**				
*No variables were entered*				
**Verbal learning**				
*Step 1*				0.21
Constant***	23.17	1.06		
SRTs in noise**	–0.50	0.15	-0.46	

*Independent variables included age, education level, aided soundfield hearing thresholds, and SRTs in noise. Education level was coded as 0 = uneducated, 1 = primary school, 2 = secondary school, and 3 = tertiary school or above.*

*HA, hearing aid; MoCA, Montreal Cognitive Assessment; SRT, speech reception threshold; B, raw coefficients; SE B, standard error of b; ΔR^2^, the change of R^2^ for each subsequent step.*

*p < 0.05, **p < 0.01, ***p < 0.001.

## Discussion

### Audiometric Thresholds Versus Speech Perception

Several previous studies reported that cognitive function weakly related to unaided audiometric thresholds which ranged from normal to severe. For example, Harrison Bush et al. (2015) reported that unaided audiometric thresholds of the better ear, measured under headphones, accounted for 0.9, 0.6–1, 0.5–1.7, and 0.4–2.2% of the variance in GCF, speed of processing, EF, and memory, respectively, in 894 older adults from the Staying Keen in Later Life study cohort. Baltes and Lindenberger (1997) reported that unaided audiometric thresholds measures under headphones accounted for 1.1% of the variance of a cognitive composite score. Additionally, Valentijn et al. (2005) found that the effect sizes of the relationship between unaided audiometric thresholds for the better ear measured under headphones and cognitive functions (e.g., verbal memory, attention, speed of information processing, cognitive flexibility) were small (*R*^2^ ≤ 0.01). However, Anstey et al. (2001) did not find a relationship between unaided bilateral audiometric thresholds measured under headphones and cognitive function. Similarly, in the current study, unaided soundfield audiometric thresholds in the UT group and aided soundfield audiometric thresholds in the HA group did not account for any variance in the evaluated cognitive functions.

In the present study, speech perception in noise alone accounted for 13–25% of the variance in GCF, WM, and EF in the UT group and 9–15% of the variance in GCF and VL in the HA group. Thus, the association between speech perception in noise and cognitive function found in the present study was much stronger than that in other studies reporting a significant relationship between audiometric thresholds and cognitive function, as discussed above. As detection of pure tones in audiometric testing depends on cochlear transduction and neuronal afferents to brainstem nuclei, and speech perception in noise involves higher auditory cortical processing taxing cognitive resources (Lin et al., 2013), it is not surprising that SRTs exhibited a stronger relationship with cognitive performance than did audiometric thresholds, especially for the ISL test, which requires an understanding of the test stimuli.

### Relationships Between Speech Perception in Noise and Cognition Functions

The finding that WM and EF were associated with speech perception in noise could be interpreted under the framework of the Ease of Language Understanding (ELU) Model (Rönnberg et al., 2013). According to this model, WM and EF could come into play when there is mismatch between perceptual input (e.g., phonology, prosody, syntax, and semantics) and the phonological representation stored in long-term memory. This mismatch is more severe in listeners with HL as background noise and HL could both introduce this mismatch (Rönnberg et al., 2019). This may explain why speech perception in noise was significantly associated with WM and EF in the UT group. However, this relationship has not been found in all listeners. For example, Füllgrabe and Rosen (2016) found that in young normal-hearing listeners, WM capacity only explained 2% of the variance in speech-in-noise identification scores. Similarly, we did not find this relationship in the HA group.

HA signal processing could also cause mismatch between perceptual input and the phonological representation, and previous studies have reported WM significantly correlated with aided speech-in-noise perception in older listeners with HL (Lunner, 2003; Foo et al., 2007; Rudner et al., 2008, 2009, 2011). However, after consistent exposure to information despite being distorted via HA, newly established and recalibrated phonological representations would gradually supplement the existing long-term memory representations in the lexicon. With the establishment of these new long-term memory representations, the role of WM/EF in speech perception would decrease (Rönnberg et al., 2013). This prediction, based on the ELU model, has been verified by Ng and Rönnberg (2020), who found that the relationship between WM and speech perception in noise decreased as HA experience increased. In the current study, HA users had 2–10 years (mean = 7.25 years) of HA use experience. Some of the participants might have already developed new long-term memory representations, decoupling the relationship between WM and speech perception in noise.

One might also argue that the same logic (i.e., acclimatization) should be applied to the UT group. That is, sentence perception in noise should not significantly associate with WM and EF in the UT group due to acclimatization. The average SNR in daily environments is approximately 5 dB (Smeds et al., 2015), which approximates the mean aided SRT in the HA group (i.e., 5.87 dB), and is much higher than the mean SRT in the UT group (2.83 dB) (Table 1). This suggests that the HA group had more opportunities and experience in practicing speech perception at an SNR close to the SRT in daily situations. The reliance on WM/EF in speech perception may thus decrease. This speculation could be verified by including another UT group with SRTs matched to those in the HA group in a future study. The lack of a relationship between WM/EF and sentence perception in noise in both groups would support this speculation.

Although aided audiometric hearing thresholds in the HA group were comparable to the unaided hearing thresholds in the UT group, the UT group was better at perceiving sentences in noise than the HA group. This may be attributed to greater suprathreshold auditory processing deficits associated with more severe HL (as observed in the HA group in the present study) (Füllgrabe and Moore, 2018). This deficit could also have affected performance in cognitive measures (Füllgrabe, 2020), although we checked to ensure participants had no problem hearing the test instructions and test stimuli. Such deficits may, thus, explain the significant relationship between speech perception in noise and VL in the HA group. Meister et al. (2013) has also reported a significant relationship between speech perception in noise and VL in older adults, and speculated that the ability to process fine structural cues may mediate the relationship. Assessment of suprathreshold processing deficits in this group in a future study could verify this speculation.

In the present study, age did not contribute to GCF, WM, EF, and SA in the UT group, and GCF, EF, SA, and VL in the HA group, when the effects of education level were controlled. These findings do not necessarily contrast with those from Western societies, for which a relationship between age and cognitive function has been reported (Naveh-Benjamin, 2000; Fisk and Sharp, 2004; Treitz et al., 2007). As mentioned above, the education level of the older Chinese adults in the current study was much lower than that of the participants in these other studies. Education level, which also was significantly related to age in the UT group, overshadow the effects of age on WM, EF, SA in the regression analyses (Tables 2, 4). As for the HA group, HA may affect the developmental trajectories of the cognitive functions and thus, altering the relationship between cognitive functions and age. This speculation could be examined using a longitudinal study with randomization of participants to the HA and UT group.

There are several limitations to the present study. First, potential confounders, such as social participation, perceived hearing difficulties, personality traits, attitudes, income, and other health aspects, and their impact on cognitive function might not have been equivalent in the two groups. Thus, the cognitive skills in the HA and UT groups were not directly compared to examine whether HA use enables older adults to retain cognitive function to a similar level as those with milder, unaided HL. To better control bias due to these confounders, a longitudinal study with a larger sample size and randomization of participants to HA and UT groups could be carried out.

Second, although the OBT scores in the HA group were slightly skewed (skewness = 0.38), those in the UT group were moderately skewed (skewness = 0.70), suggesting that the OBT was relatively easy for this group, and accordingly, the relationship between WM and speech perception in noise might have been obscured.

Third, the current study included a wide range of HA brands and models. HA features (e.g., compression parameters, noise reduction, and directional microphone) were not included in the analysis. As some HA features (e.g., compression parameters) have been found to be significantly related to speech perception in noise (Stone et al., 2009b; Chen et al., 2021), it is possible that these HA features might have mediated the relationship between cognitive function and sentence perception in noise in the HA group.

Fourth, there was no significant difference in audibility (aided soundfield hearing thresholds in the HA group and unaided soundfield hearing thresholds in the UT group) between the HA and UT groups. Whether these results could generalize to other populations, in which audibility between UT group and HA groups differ, requires further study.

Finally, although a relationship between speech perception and cognitive function was established in the current study, little is known about the causality or underlying mechanisms of this relationship. The current study has demonstrated which cognitive functions are more likely associated with HL in UT and HA populations, and this information could be used to understand the potential mechanisms underlying auditory sensory and cognitive functions in future studies.

## Data Availability Statement

The raw data supporting the conclusions of this article will be made available by the authors, without undue reservation.

## Ethics Statement

The studies involving human participants were reviewed and approved by Human Research Ethics Committee at the University of Hong Kong (EA1706014) and the Joint Chinese University of Hong Kong—New Territories East Cluster Clinical Research Ethics Committee (CRE-2013.481). The patients/participants provided their written informed consent to participate in this study.

## Author Contributions

SSC and JY collected the data. LW, YC, JY, and SSC contributed to the study design. LW, YC, and SSC interpreted, wrote, and reviewed this publication. All authors approved the final manuscript.

## Conflict of Interest

The authors declare that the research was conducted in the absence of any commercial or financial relationships that could be construed as a potential conflict of interest.

## Publisher’s Note

All claims expressed in this article are solely those of the authors and do not necessarily represent those of their affiliated organizations, or those of the publisher, the editors and the reviewers. Any product that may be evaluated in this article, or claim that may be made by its manufacturer, is not guaranteed or endorsed by the publisher.

## Footnotes

1
http://www.Cogstate.com

2
http://www.mocatest.org/

## Abbreviations

EF: executive function
GCF: general cognitive function
HL: hearing loss
HA: hearing aid
SRT: speech recognition threshold
CHINT: Cantonese Hearing in Noise Test
NF: noise front
NBE: noise better ear
NEW: noise worse ear
SNR: signal-to-noise ratio
SA: sustained attention
MoCA: Montreal Cognitive Assessment
CSB: CogState Battery
MCI: mild cognitive impairment
OBK: One-Back Test
GML: Groton Maze Learning Test
IDN: Identification Test
ISL: International Shopping List Task
SD: standard deviation
PTA: pure tone average
UT: untreated
VL: verbal learning
WM: working memory.
